# OGT and OGA: Sweet guardians of the genome

**DOI:** 10.1016/j.jbc.2024.107141

**Published:** 2024-03-04

**Authors:** Chen Wu, Jiaheng Li, Lingzi Lu, Mengyuan Li, Yanqiu Yuan, Jing Li

**Affiliations:** 1 College of Life Sciences, Institute of Life Sciences and Green Development, Hebei University, Baoding, Hebei, China; 2 School of Pharmaceutical Sciences, Guangdong Provincial Key Laboratory of Drug Non-Clinical Evaluation and Research, Sun Yat-sen University, Guangzhou, Guangdong, China; 3 Beijing Key Laboratory of DNA Damage Response and College of Life Sciences, Capital Normal University, Beijing, China

**Keywords:** O-GlcNAc, OGT, OGA, DNA damage, replication

## Abstract

The past 4 decades have witnessed tremendous efforts in deciphering the role of O-GlcNAcylation in a plethora of biological processes. Chemists and biologists have joined hand in hand in the sweet adventure to unravel this unique and universal yet uncharted post-translational modification, and the recent advent of cutting-edge chemical biology and mass spectrometry tools has greatly facilitated the process. Compared with O-GlcNAc, DNA damage response (DDR) is a relatively intensively studied area that could be traced to before the elucidation of the structure of DNA. Unexpectedly, yet somewhat expectedly, O-GlcNAc has been found to regulate various DDR pathways: homologous recombination, nonhomologous end joining, base excision repair, and translesion DNA synthesis. In this review, we first cover the recent structural studies of the O-GlcNAc transferase and O-GlcNAcase, the elegant duo that “writes” and “erases” O-GlcNAc modification. Then we delineate the intricate roles of O-GlcNAc transferase and O-GlcNAcase in DDR. We envision that this is only the beginning of our full appreciation of how O-GlcNAc regulates the blueprint of life—DNA.

O-GlcNAcylation, the quintessential glycosylation that occurs intracellularly, is catalyzed by the sole O-GlcNAc transferase (OGT) and removed by the only glycosidase O-GlcNAcase (OGA). Discovered exactly 4 decades ago, O-GlcNAcylation mediates various cellular processes, including transcription, translation, autophagy, and immune response (1, 2). Its aberrancy leads to many human diseases, such as cancer, cardiovascular diseases, and neurodegenerative diseases. Mechanistically, O-GlcNAcylation frequently crosstalks with other post-translational modifications (PTMs), such as phosphorylation, acetylation, ubiquitination, and recently poly(ADP-ribosyl)ation (PARylation), either by regulating the “writer” or “eraser” enzymes or by directly competing for the same amino acids on the substrate proteins. One of the most frequently studied examples is the crosstalk between O-GlcNAcylation and phosphorylation. Ser residues or Thr residues on the same protein may be modified by either O-GlcNAcylation or phosphorylation, creating the potential for competition at the same or proximal sites.

During the cell cycle, cells are exposed to various internal and external hazards. Internal DNA damage primarily occurs because of hydrolysis and oxidation of chemically active DNA by water and intracellular reactive oxygen species (ROS). External DNA damage, on the other hand, is caused by environmental, physical, and chemical agents such as UV and ionizing radiation (IR), alkylating agents, and crosslinking agents. These hazardous factors lead to different types of DNA damage, including abasic sites, mismatches, interstrand crosslinks (ICLs), single-strand breaks (SSBs), and double-stranded breaks (DSBs) (3). Among these lesions, DSBs are particularly harmful and pose a significant threat to cells. To counteract these damages, cells have evolved DNA damage response (DDR) pathways that identify and repair specific types of DNA lesion, ensuring the integrity of the genome. Dysregulation of DDR results in chromosome instability and eventually lead to tumorigenesis (4). The DDR pathways include homologous recombination (HR), nonhomologous end-joining (NHEJ), mismatch repair, base excision repair (BER), nucleotide excision repair, and ICL repair (5, 6). These repair processes are crucial for maintaining genome stability (7).

Recent years, emerging evidence has implicated O-GlcNAcylation in DDR. Here, we first review the recent structural insights of OGT and OGA, as scientists strive to answer why there is only one enzyme for both writing and erasing O-GlcNAc. The advent of cryo-EM surely brought new insights into the question but still left many issues unanswered. Then we provide a comprehensive review of the current understanding of the role of O-GlcNAcylation in DDR, highlighting its impact on DNA replication, DNA repair pathways, and genome integrity.

## Structural insights into OGT and OGA substrate specificity

OGT is responsible for catalyzing the transfer of O-GlcNAc from cellular UDP-GlcNAc onto Ser residues or Thr residues of a myriad of cytoplasmic and nuclear proteins, forming β-linked O-GlcNAc additions (8, 9), whereas OGA is responsible for their removal (10).

### The catalytic mechanism of OGT

**Figure 1:**
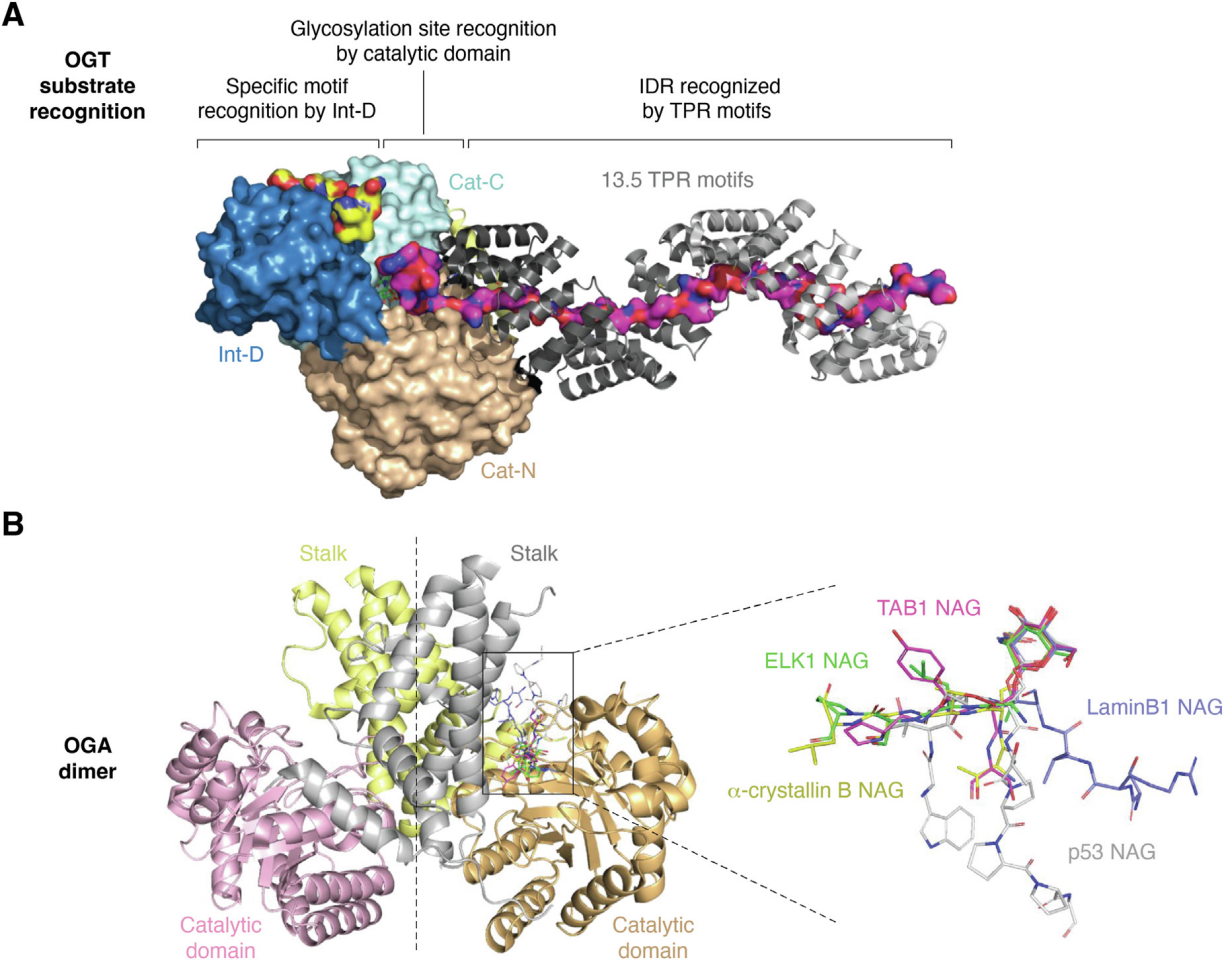
**Structures of OGT and OGA.***A*, a schematic model for OGT substrate recognition mechanisms. The recognition involves glycosylation site selection by the catalytic domain, potential motif recognition by the Int-D, and/or engagement from the long and extended TPR motifs. The model was generated by the alignment of the EM structure of OGT in complex with OGA (Protein Data Bank [PDB] code: 7YEH) and a structure of OGT in complex with a peptide from SMG9 (PDB code: 8FE7) using PyMOL 2.3.0. The N- and C-terminal parts of the OGT catalytic domain (Cat-N and Cat-C) and the Int-D are shown in surface representation and colored in *light orange*, *pale cyan*, and *blue*, respectively. The TPR motifs are shown in *cartoon representation* and colored in *gray*. The C-terminal fragment of OGA and SMG9 peptide is shown in surface representation with carbon atoms shown in *magenta* and *yellow*, respectively. UDP in the active site is shown in a *stick representation* (carbon atoms in *green*). *B*, a structural model for OGA and its peptide substrates. The model was generated by the alignment of OGA structures (the D175N mutant compassing the catalytic domain and the stalk region) in complex with glycopeptide substrates derived from the following proteins: P53 (PDB code: 5UN8), α-crystallin B chain (PDB code: 5VVV), TAB1 (PDB code: 5VVU), ELK1 (PDB code: 5VVT), and Lamin B1 (PDB code: 5VVX) using PyMOL 2.3.0. In the *left panel*, the catalytic domain and stalk region of the OGA dimer are depicted in a *cartoon representation*, colored in *light orange* (*light pink*) and *gray* (*pale yellow*), respectively. The *right panel* provides a close-up view of the overlaid glycopeptides in a *stick representation*, with carbon atoms depicted using the same color as their labels. Int-D, intermediate domain; OGA, O-GlcNAcase; OGT, O-GlcNAc transferase; TPR, tetratricopeptide repeat.

Since the initial discovery of OGT, researchers have identified thousands of proteins that undergo O-GlcNAc modification, leading to extensive efforts to understand how OGT recognizes its substrates. Structurally, OGT contains spiral-shaped tetratricopeptide repeat (TPR) domains that precede its N-terminal catalytic (N-Cat) and C-terminal catalytic (C-Cat) domains (11, 12, 13). Both N-Cat and C-Cat domains contain Rossman folds, unique to the glycosyltransferase type B family. But there is also an additional intermediate domain (Int-D) between N-Cat and C-Cat domains, which is not commonly observed in the glycosyltransferase type B family (Fig. 1) (14, 15).

The catalytic mechanism of OGT has been under intense study in the last few decades and has been well reviewed previously (16, 17, 18, 19, 20, 21). Briefly, it is supported by both structural and kinetic studies that the catalytic domain of OGT utilizes a metal-independent glycosyl-transfer mechanism where it first binds the sugar donor followed by protein substrate binding (15, 16, 22). The inversion of the anomeric configuration of the transferred GlcNAc occurs through nucleophilic attack by the sugar recipient (16). However, the precise deprotonation mechanism and the involvement of auxiliary elements in this process have yet to be resolved (17, 18).

In the crystal structures of OGT complexed with peptide substrates, all peptides are found to adopt an extended conformation within the shallow binding cleft of the OGT active site. However, few specific interactions involving peptide side chains could be identified to contribute to the association (14). Instead, it appears that a combination of size and restricted torsion angles plays a crucial role in defining the sequence preference near the glycosylation site.

It is important to note that the peptide sequence surrounding the modification site is not the sole determinant of substrate recognition by OGT. Structural data indicate that the peptide substrates bound to OGT are consistently oriented in the same direction, with the C-terminal ends extending toward the TPR region (15, 16, 17, 23, 24). In the complex structure of OGT and the host cell factor 1 fragment, the peptide establishes specific contact with conserved Asn residues that line the inner surface of the concave formed by interconnected TPR domains (24). Subsequent protein microarray and proteomic profiling revealed that the majority of OGT substrates were recognized by OGT *via* the Asn and Asp ladders in the TPR lumen proximal to the catalytic domain (24, 25, 26, 27). In a recent cryo-EM image capturing the OGT and OGA complexes, where OGA served as a substrate for OGT, it was observed that a GlcNAc moiety is covalently attached to the hydroxyl group of Ser-405 in OGA (19), representing a postreaction binding mode. The OGA residues following Ser-405 adopt an extended conformation, protruding from the catalytic center of OGT and occupying the entire substrate-binding lumen of the TPR domain. These interactions are mediated by a network of hydrogen bonds involving the peptide backbones. These studies have provided evidence that TPR domains are likely the key players in OGT substrate recognition, and the interactions are mediated by sequence-independent interactions with long flexible regions located after the sugar acceptor residue, as is the case for the intrinsically disordered regions in OGA. This study also left us with one open question: why is OGT in complex with OGA, if one is the writer and the other is the eraser? How can they coordinate their actions to be energetically efficient?

Apart from TPR, some other factors are found to be involved in OGT substrate recognition. Notably, two publications highlighted the biological importance of the Int-D of OGT in substrate selectivity (28, 29). Using peptide phage display, both studies enriched a PxYx[I/L/M/F] motif in OGT binding *via* the Int-D. Importantly, this motif could be identified within the intrinsically disordered regions of O-GlcNAcylated proteins in the human proteome, substantiating the physiological relevance of this motif in OGT binding.

### Proteomic profiling of OGT substrates

A wealth of cytoplasmic and nuclear substrates have been identified through the application of various chemical biology and mass spectrometry strategies (30, 31, 32, 33, 34, 35, 36, 37, 38, 39, 40, 41, 42, 43, 44, 45, 46, 47, 48). In 2020, Eugenia *et al.* (49)conducted a systematic analysis of all published articles on human O-GlcNAc at that time, resulting in an extensive inventory of 5072 human O-GlcNAcylated proteins and 7002 O-GlcNAc sites (www.oglcnac.mcw.edu), and later, another database on O-GlcNAc sites became publicly available (www.oglcnac.org) (50). The compiled list presents a comprehensive overview of O-GlcNAc modification in the human proteome. By conducting an analysis of the amino acid sequences flanking the O-GlcNAc sites, it was concluded that OGT does not have preference between Ser residues and Thr residues as glycosites. While O-GlcNAc sites do not have a strict consensus, the semiconsensus sequence was refined as follows: P-P-(V/T)-g(S)-(S/T)-A and (P/T)-P-(V/T)-g(T)-(S/T)-(A/T), which is in broad agreement with biochemical and structural findings (23). The meta-analysis also confirmed that O-GlcNAcylation modulates the binding affinity of proteins to RNA–DNA, influencing their participation in RNA metabolism and thereby exerting control over genomic information. There are additional dozens of publications on human O-GlcNAc proteomic profiling since 2020 (37, 38, 39, 40, 41, 43, 44, 45, 46, 47, 48) however, the conclusion regarding substrate sequence preference remains the same.

### Structure and substrate recognition of OGA

As nutrient sensors in human cells, O-GlcNAc levels are tightly controlled through the coordinated activities of OGT and OGA (11, 51, 52, 53). Evidence has demonstrated that the expression levels of OGT and OGA exhibit rapid responses to changes in OGT activity (54, 55, 56, 57), highlighting the essentiality of precise control over O-GlcNAc levels. As the sole known glycosidase for removing protein β-O-GlcNAc modifications in the cell, the activity of OGA has been the subject of extensive evaluation and scrutiny.

OGA consists of an N-terminal catalytic domain and a C-terminal pseudo histone acetyltransferase (HAT) domain, which are connected by a stalk domain (10). The catalytic activities of the HAT-like domain have not been confirmed, and its role in OGA remains poorly understood. By overlapping the three available human OGA structures, despite variations in construct designs and crystallization strategies, the GlcNAc molecules of the glycopeptide substrates align perfectly in the active site and establish a network of interactions with active sites of OGA, underscoring the essential role of O-GlcNAc binding in substrate recognition by OGA (58, 59, 60).

OGA exists as a stable dimer in solution (59, 60), and the substrate binding cleft is formed between the catalytic domain and the stalk region of the sister monomers. While hydrophobic residues are favored at certain positions in the substrate peptide, for example, W, at the −3 position (59, 61), it is noteworthy that the binding mode of different peptides and even the same peptide in the two monomers can vary significantly (58, 59, 60, 62). In addition, the peptide backbones can be oriented in opposite directions (58, 62), signifying a highly permissive substrate selection in the vicinity of the glycosite. Accordingly, the kinetic parameters (*k*_cat_/*K*_*m*_) of OGA for a limited set of protein substrates were not significantly different (22), further supporting the notion of OGA’s broad substrate specificity.

Importantly, the catalytic efficiency of OGT and OGA may not be the same for different protein substrates, and additional factors may contribute to the maintenance of the O-GlcNAc levels on specific proteins, such as PTMs, cellular localizations, as well as interaction partners. In addition, OGT and OGA are also found in other eukaryotes but not always in pairs. In plants, there is Secret Agent (Sec), which is the OGT homolog. But no OGA has been identified so far. In *Drosophila*, while both OGT and OGA are present, only OGT is essential (63). Further in-depth investigations of OGT and/or OGA activity in various organisms may provide valuable insights into O-GlcNAc regulatory mechanisms.

## Impact of O-GlcNAcylation on replication

**Figure 2:**
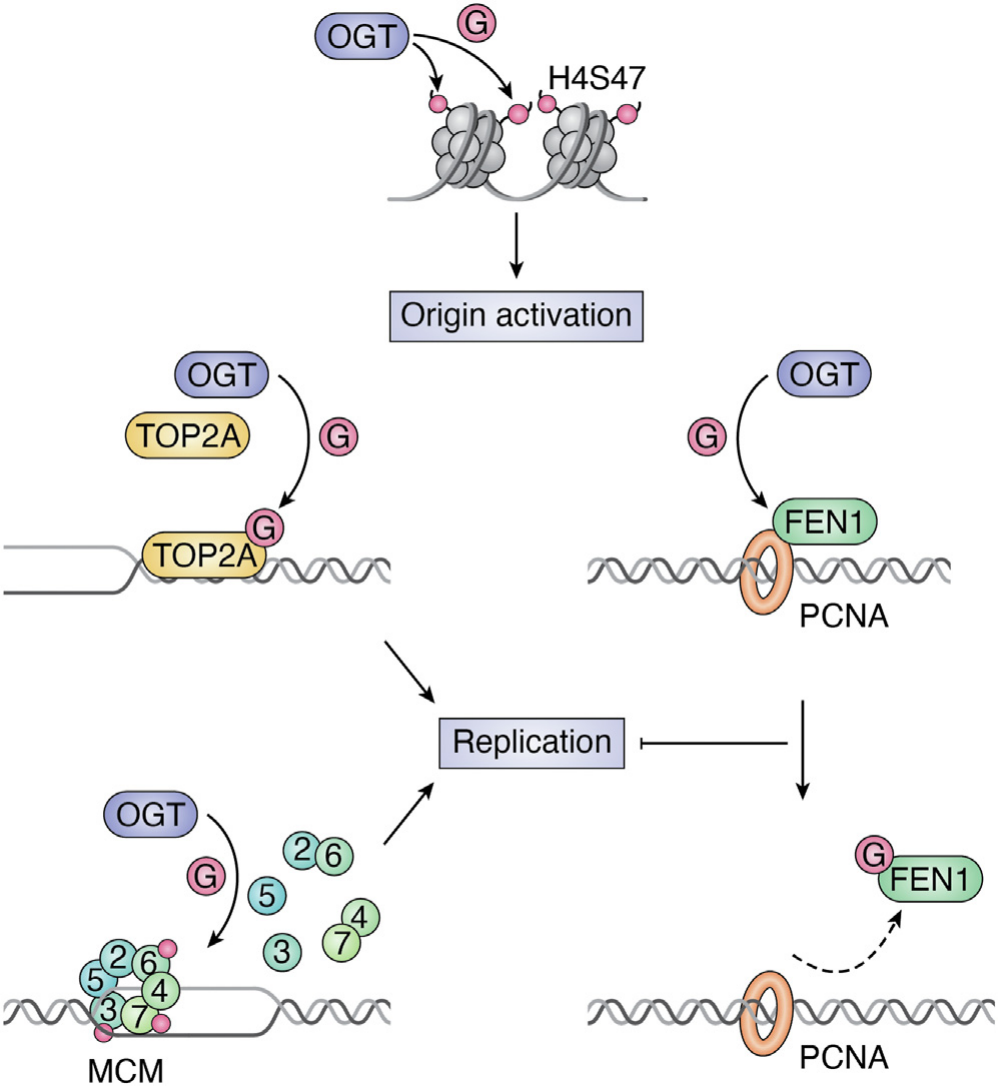
**The role of OGT in DNA replication.** During DNA replication, proliferating cell nuclear antigen (PCNA) functions as a “sliding clamp” to facilitate the positioning of proteins on DNA. One crucial protein involved in DNA replication is the endonuclease FEN1. O-GlcNAcylation modification of FEN1 weakens its interaction with PCNA, thereby impairing DNA replication. Conversely, O-GlcNAcylation modification of topoisomerase TOP2A enhances its activity and DNA-binding ability, facilitating changes in DNA conformation. The assembly of the MCM2–7 complex on DNA plays a critical role in initiating DNA replication. O-GlcNAcylation modification of the MCM2, 3, 6, and 7 subunits promotes the stability of the interactions between MCM2/6 and MCM4/7. Furthermore, O-GlcNAcylation of histone H4 at position S47 also plays a significant role in activation of DNA replication origins in response to DNA replication stress. This modification is involved in coordinating the cellular response to DNA damage and ensuring the appropriate timing and control of DNA replication under stressful conditions. FEN1, flap endonuclease 1; OGT, O-GlcNAc transferase; TOP2A, topoisomerase IIα.

Accurate replication of the genome is crucial to ensure the correct transmission of genetic information to daughter cells during each round of cell division. Errors in replication can lead to mutations and chromosomal abnormalities, which result in genomic instability and tumorigenicity. It is not surprising that O-GlcNAcylation plays a crucial role during DNA replication (Fig. 2).

The early findings come from studying the minichromosome maintenance (MCM) proteins. In human cells, the MCM ring comprises a hexamer of Mcm2–7, which is important for DNA replication licensing, that is, one and only one round of DNA replication per cell cycle. The hexameric MCM helicases facilitate DNA unwinding and replication initiation and gradually dissociate from chromatin in late S phase. Leturcq *et al.* (64) found that the subunits of the MCM2–7 complex are O-GlcNAcylated, which mainly occurs in the chromatin-bound fraction of synchronized human cells. OGT binds to and modifies the MCM2, 3, 6, and 7 subunits, increasing the stability of MCM–MCM ring interaction and enhancing their binding to chromatin. Because of the limitations of this study, the modified sites are left unfound.

Recently, OGT is found to be enriched at DNA replication origins during the S phase and enhances the activation of DNA replication initiation by catalyzing O-GlcNAcylation of histone H4S47. When the H4S47 residue is mutated to Ala, its O-GlcNAcylation level is significantly reduced, leading to a notable inhibition of origin density (65). H4S47 O-GlcNAcylation regulates replication initiation by enhancing interaction with the MCM ring. Moreover, it promotes the phosphorylation of MCM2 at Ser-53 and the complex formation among H4, Cdc7–Dbf4 kinase (DDK), and MCM, which is crucial for replication initiation (65).

Topoisomerase IIα (TOP2A) is responsible for untangling the double helical structure of DNA, providing the necessary single-stranded template for replication, and is also an important target for anticancer drugs. Liu *et al.* (66) found that elevated TOP2A, especially its O-GlcNAcylation, promotes malignant progression in breast cancer and resistance to adriamycin. OGT mediates TOP2A O-GlcNAcylation at Ser-1469, thereby enhancing the binding of TOP2A to chromatin and promoting its topoisomerase activity. Disrupting O-GlcNAcylation of TOP2A significantly enhances the therapeutic effect of adriamycin in xenograft models. Mechanistically, O-GlcNAcylation modulates interactions between TOP2A and cell cycle regulators, influences downstream gene expression, and contributes to breast cancer drug resistance. This study provided support for targeting TOP2A O-GlcNAcylation in cancer therapy.

Moreover, human flap endonuclease 1 (FEN1) is also O-GlcNAcylated. Fen1 is a structure-specific and multifunctional endonuclease essential for DNA replication and repair. A previous study showed that FEN1 undergoes small ubiquitin-related modifier modification (SUMOylation) in response to DNA replication fork-stalling agents, such as UV, hydroxyurea, and mitomycin C, which promotes the interaction of FEN1 with the Rad9–Rad1–Hus1 complex and DNA damage repair (67). Recently, Tian *et al.* (68) revealed an important role of O-GlcNAcylated FEN1 in regulating DNA replication and repair. FEN1 O-GlcNAcylation at Ser-352 is dynamically regulated during the cell cycle. It not only disrupts the interaction of FEN1 with proliferating cell nuclear antigen at the replication foci but also leads to altered cell cycle, defects in DNA replication, accumulation of DNA damage, and enhanced sensitivity to DNA damage agents (68). By virtue of the enrichment of OGT in the nucleus, it is reasonable to speculate that there are many more OGT substrates involved in DNA replication.

## OGT and OGA in DDR

PTMs play crucial roles in various DDR pathways. When cells experience DNA damage, a series of PTMs, including phosphorylation, ubiquitination, O-GlcNAcylation, and ADP-ribosylation, are activated. These modifications, in turn, regulate the biological functions of various DNA damage repair factors (69, 70, 71, 72). Accumulating evidence has also demonstrated that O-GlcNAcylation, like other modifications, is induced by DNA damage (73, 74, 75). We, together with other investigators, have shown that both OGT and OGA are recruited to the sites of DNA damage, facilitating reversible O-GlcNAcylation of proteins at DNA lesions (73, 76). In addition, exposure to UV enhances the chromatin binding of OGT, which subsequently influences the cellular response to UV (Table 1) (75).

**Table 1:** The effect of OGT and OGA on DDR pathways

DDR pathways	OGT/OGA localization, protein abundance, and overproduction/knockdown studies	References
HR and single-strand annealing (SSA)	OGT deletion or inhibition analysis shows that OGT is essential for Rad52 IRIF formation. OGT is not required for chromosomal break end joining or Rad51 IRIF formation	(85)
DSB repair	In MCF7 human mammary carcinoma cells and xenograft tumors, upregulating of O-GlcNAc protected tumor xenografts against radiation. Downregulating O-GlcNAc delayed DSB repair, reduced cell proliferation, and increased cell senescence	(110)
DDR	In fly stem/progenitor cells, in mouse embryonic stem cells (ESCs) and mouse embryonic fibroblasts (MEFs), CHK1/2 stabilize OGT, augmenting O-GlcNAcylation and further promoting DDR	(81)
NHEJ	OGA is recruited to DNA lesions by its C-terminal pseudo-HAT domain. OGA suppression impaired NHEJ	(76)
HR	OGT is recruited to DNA damage sites	(73)
DDR-related pathways	Ataxia telangiectasia mutated (ATM) inhibition in the ovarian cancer cell line SKOV3 abnormally elevated OGT and OGA levels	(111)
UV damage	Chromatin binding of OGT was enhanced after UV treatment. OGT affects cellular response to UV radiation	(75)

**Figure 3:**
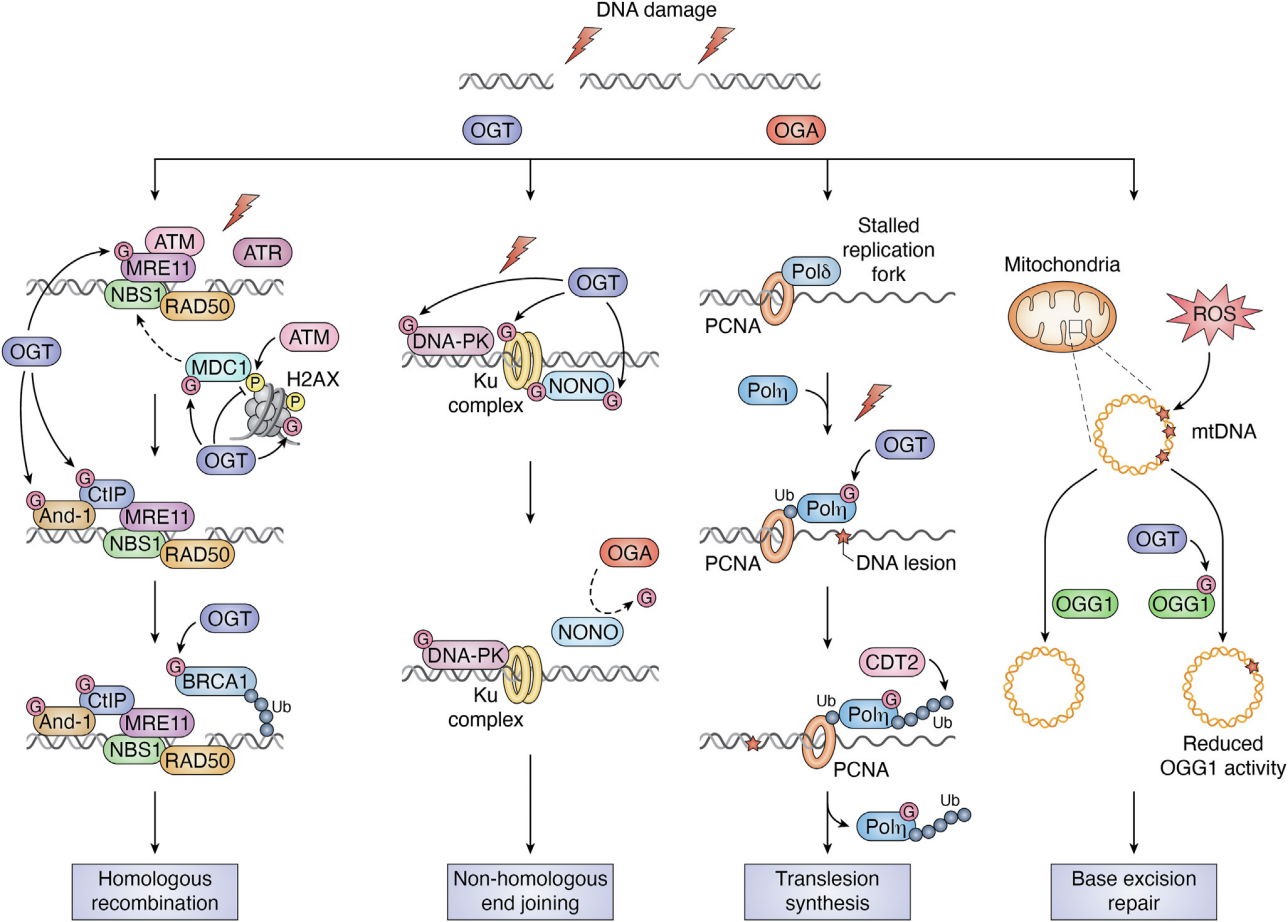
**The role of OGT and OGA in DNA damage response.** When DNA double-strand breaks (DSBs) occur, OGT is recruited to DNA damage sites. It O-GlcNAcylates H2AX and MDC1, which leads to the inhibition of γ-H2AX-mediated phosphorylation events. In the homologous recombination (HR) repair pathway: MRN complexes are recruited to DNA damage sites, and O-GlcNAcylation enhances MRE11 activity and binding strength to chromatin. CtIP and And-1, responsible for DNA end excision, are recruited to DNA damage sites by MRE11. O-GlcNAcylation of CtIP and And-1 enhances their chromatin-binding ability and improves the efficiency of DNA end excision during HR. In the nonhomologous end joining (NHEJ) repair pathway: after DNA DSBs, the Ku70–80 complex rapidly assembles and binds rapidly to the DNA end, recruiting and activating DNA-PKcs and NONO. OGT modifies DNA-PKcs, Ku70/80, and NONO in response to DNA damage. Inhibition of OGA activity prolongs the O-GlcNAcylation of Ku70/80 and NONO at the DNA damage site, thereby extending the recruitment of NONO and inhibiting the NHEJ repair pathway. In the translesion synthesis (TLS) repair pathway: UV and cisplatin treatment causes replication forks to stall. Polη is recruited to the stalled replication forks and undergoes O-GlcNAcylation by OGT. After completing TLS, Polη is polyubiquitinated. It is dissociated from the replication forks and degraded. In the base excision repair (BER) pathway: oxidative stress–induced damage to mitochondrial DNA (mtDNA) often leads to the formation of 8-hydroxy-2′-deoxyguanosine (8-OHdG) at the DNA base. O-GlcNAcylation of OGG1 reduces its enzymatic activity, thereby limiting the excision of 8-OHdG by OGG1 during BER. BER, base excision repair; DNA-PKcs, DNA-PK catalytic subunit; OGA, O-GlcNAcase; OGT, O-GlcNAc transferase; MRN, MRE11–RAD50–NBS1; NONO, non-POU domain–containing octamer-binding; Polη, polymerase η.

Because of the transient nature of DDR, new chemical tools are developed to examine the spatial and temporal control of O-GlcNAc. In 2011, Zachara *et al.* (77)highlighted the function of O-GlcNAc in regulating DDR and other cellular pathways. Chemists also used a triarylphosphine–trimethylpiperidine reagent for enrichment of O-GlcNAcylated proteins after IR (78). Furthermore, a highly sensitive one-step enzymatic strategy was developed to visualize O-GlcNAcylated proteins during DNA damage (68). A recent study has also shown that O-GlcNAcylation affects the pathway choice of DSB repair (79). Here, we dissect the role of O-GlcNAcylation in different repair pathways (Fig. 3 and Tables 1 and 2).

**Table 2:** OGT and OGA substrates in DDR

Biological processes	Protein name	Molecular details	References
Replication	MCM	Mcm3,6,7 are O-GlcNAcylated	(64)
Replication	Histone 4 (H4)	H4 is O-GlcNAcylated at Ser47. H4S47 O-GlcNAcylation enhances DBF4-dependent protein kinase (DDK) recruitment on chromatin, directs origin activation through facilitating MCM phosphorylation	(65)
Replication	TOP2A	O-GlcNAcylation at Ser1469 enhances TOP2A chromatin DNA binding and catalytic activity, leading to resistance to Adm in breast cancer cells and xenograft models	(66)
Replication	Flap endonuclease 1 (FEN1)	FEN1 is O-GlcNAcylated at Ser352, which disrupts its interaction with proliferating cell nuclear antigen (PCNA) at the replication foci, and leads to altered cell cycle, defects in DNA replication, accumulation of DNA damage, and enhanced sensitivity to DNA damage agents	(68)
TLS	Polη	Polη is O-GlcNAcylated at T457, which promotes polyubiquitination at K462 and subsequent removal from replication forks	(75)
HR	Mediator of DNA damage checkpoint protein 1 (MDC1)	MDC1 is O-GlcNAcylation during DDR O-GlcNAcylation of MDC1 is increased upon irradiation	(73) (79)
HR	CtIP, BRCA1	CtIP and BRCA1 became increasingly O-GlcNAcylated after irradiation The chromatin underwent reorganization upon modulating O-GlcNAcylation	(79)
HR	AND-1	AND-1 is O-GlcNAcylated at S575 and S893, which affects the recruitment of AND-1 and CtIP to DDR sites and regulates radioresistance in colorectal cancer cells	(112)
HR	YTHDC1	YTHDC1 is O-GlcNAcylated at S396 upon DDR, which promotes recruitment of YTHDC1 to DDR sites and YTHDC1–m^6^A binding. It enhances YTHDC1–m^6^A condensate formation, Rad51 focus formation, and HR	(88)
HR and NHEJ	Histone H2B	H2B S112 O-GlcNAcylation increased upon DSB, which promotes HR and NHEJ by binding with NBS1 and enhances NBS1 IRIF formation	(83)
NHEJ	DNA-PK	DNA-PKcs O-GlcNAcylation promotes its kinase activity and antagonizes bleomycin-induced Ser2056 phosphorylation	(91)
NHEJ	NONO and Ku70/80	NONO and Ku70/80 are recognized by the pseudo HAT domain of OGA for deglycosylation, which is required for NONO dissociation from the chromatin and degradation	(76)
CDK9 inhibitor–induced DNA damage in castration-resistant prostate cancer (CRPC)	Mre11	OGT and MRE11 are essential for the repair of CDK9 inhibitor–induced DNA damage. Mechanistically, OGT is required for MRE11 chromatin loading in cells treated with CDK9 inhibitor. MRE11 and O-GlcNAc are enriched at the prostate cancer–specific small nucleotide polymorphic sites	(84)
Error-prone DDR pathway	HMGB1	HMGB1 is O-GlcNAcylated at S100 in human non–small-cell lung carcinoma cells. O-GlcNAc reduces the ability of HMGB1 to facilitate DNA repair, resulting in error-prone processing of damaged DNA. Mechanistically, O-GlcNAc enhances HMGB1 oligomerization on linear, nucleosomal, supercoiled, cruciform, and interstrand cross-linked damaged DNA structures	(105)

### O-GlcNAcylation in HR

Increasing evidence supports the notion that O-GlcNAcylation plays a crucial role in enhancing DSB repair and promoting cancer cell proliferation. Inhibition of O-GlcNAcylation through the blockade of OGT activity has been demonstrated to delay DSB repair, reduce cell proliferation, and heighten cellular senescence *in vivo* (76). We showed that checkpoint kinase 1 phosphorylates and stabilizes OGT at Ser-20 during cytokinesis (80). Later, both checkpoint kinase 1/2 are found to stabilize OGT, thereby increasing O-GlcNAcylation and further augmenting DDR in mouse embryonic stem cells and mouse embryonic fibroblasts (80, 81).

O-GlcNAcylation plays an important role in HR. One OGT-modified histone is γH2AX, a marker of DNA damage. Chen *et al.* (73) discovered that various types of DNA damage induction enhance the O-GlcNAcylation of chromatin components. The amino acid 462 to 510 fragment of OGT, which connect the N-terminal TPR and C-terminal catalytic domains, serves as the key motif for recruiting OGT to DNA damage sites. Furthermore, O-GlcNAcylation of γH2AX at Ser-139 inhibits its phosphorylation and acts as a negative regulator, limiting the expansion of γH2AX at DNA damage sites (73). Sustained phosphorylation of H2AX leads to G2/M arrest and apoptosis, whereas O-GlcNAcylation of γH2AX inhibits local DNA damage and aids DNA repair. Meanwhile, inhibiting OGT enzyme activity significantly reduces cellular resistance to IR (73). Under basal conditions, H2AX is found to be O-GlcNAcylated at Thr-101, but its function is left unexplored (73). Moreover, H2A is found to be O-GlcNAcylated at Ser-40 upon camptothecin treatment or etoposide treatment, which facilitates DNA damage (82).

The MRE11–RAD50–Nijmegen breakage syndrome 1 (NBS1) (MRN) complex plays a critical role in recognizing damage sites and initiating HR for DNA repair. Wang *et al.* (83) found that O-GlcNAcylated histone H2B interacts with NBS1, a component of the MRN complex. The enhanced interaction between O-GlcNAcylated histone H2B and NBS1 regulates the recruitment of NBS1 to DNA damage sites (83), which is a key protein that stabilizes the MRN complex at DNA damage sites (Fig. 3). Direct inhibition of OGT activity significantly reduces the recruitment of NBS1 to DNA lesions. Similar conclusions were drawn by Averbeck *et al.* (79)showing that inhibiting OGT accelerates the dissociation of NBS1 from DNA damage sites. The endonuclease activity of MRE11 is responsible for processing DSB ends. Gondane *et al.* (84)reported that O-GlcNAcylation of MRE11 promotes its binding to chromatin, and the activity of MRE11 is regulated in response to CDK9 inhibition in an OGT-dependent manner. It is known that OGT is rapidly recruited to DNA damage sites and increases O-GlcNAcylation in the vicinity of damaged DNA (73). This suggests that O-GlcNAcylated MRE11 may potentially play a role in DDR.

Furthermore, a study conducted by Ping *et al.* (85) discovered that OGT plays a significant role in RAD51-dependent homology-directed repair and single-strand annealing. The disruption of OGT had a noticeable impact on the recruitment of RAD52 into IR-induced foci (IRIF), but not RAD51 IRIF, the latter being a well-known player of DNA HR repair. This suggests that OGT is crucial for the regulation of homology-directed repair, which is partially associated with RAD52 function.

Other O-GlcNAcylated DSB factors include And-1, important for end resection of HR. And-1 interacts with OGT and is O-GlcNAcylated. O-GlcNAcylation of And-1 was detected as early as 5 min after IR, and this modification is necessary for the recruitment of C-terminal binding protein (CtBP)－C-terminal interacting protein CtIP (a cofactor for the MRN complex) to DNA damage sites for efficient DNA end resection (86). Interestingly, Averbeck *et al.* (79)used O-GlcNAc modification–specific antibodies to immunoprecipitate lysates from irradiated HeLa cells and found the presence of key proteins in HR, including CtIP, breast cancer susceptibility gene 1 (BRCA1), replication protein A (RPA), RAD51, and DNA damage checkpoint 1 (MDC1). O-GlcNAcylation of MDC1, CtIP, and BRCA1 was increased upon irradiation (79). But neither RAD51 nor RPA was detected in the O-GlcNAcylated protein fraction, suggesting that only a portion of the DDR factors are O-GlcNAcylated. It is also plausible that these proteins are O-GlcNAcylated at different temporal order.

YTH domain containing 1 (YTHDC1) is an m^6^A reader that has been shown to be recruited to the DNA–RNA hybrid at DNA damage sites and regulate HR (87). A recent chemoproteomic screen identifies YTHDC1 O-GlcNAcylation upon IR treatment (78). Our work first confirmed that YTHDC1 is O-GlcNAcylated at Ser-396 upon IR, which promotes recruitment of YTHDC1 to DNA lesions and binding between YTHDC1 and m^6^A mRNA. It also enhances YTHDC1–m^6^A condensate formation for phase separation, Rad51 IRIF formation, and HR (88), which is consistent with previous findings that HR repair is affected by OGT.

### OGA in NHEJ

O-GlcNAcylation also plays an important role in NHEJ, which is the predominant form for DSB repair during the cell cycle. But it can generate insertions and deletions. DNA-dependent protein kinase (DNA-PK) is a key player of NHEJ. The DNA-PK catalytic subunit (DNA-PKcs) has multiple phosphorylation sites in clusters, some of which are autophosphorylated after DNA damage (89, 90). For example, the phosphorylation of Ser-2056 regulates the conformation and activity of DNA-PKcs and has an essential role in impacting DSB end processing and NHEJ (90). Lafont *et al.* (91) showed an antagonistic effect between DNA-PKcs O-GlcNAcylation and its autophosphorylation at Ser-2056 during bleomycin-induced DNA damage. However, an elevation of O-GlcNAcylation does not affect DNA-PKcs activity, whereas its kinase activity is affected by a decrease of O-GlcNAcylation, suggesting that an O-GlcNAcylation and phosphorylation interplay fine-tunes DNA-PKcs activity to maintain genome integrity (91).

We demonstrated that the C-terminal HAT domain of OGA recognizes early DSB response factors for deglycosylation, such as non-POU domain–containing octamer-binding (NONO) (76). It is known that NONO is recruited to DNA damage sites and promotes NHEJ (92, 93). We discovered that OGA is also recruited to DNA damage sites *via* its HAT domain (although the kinetics is slower than OGT) (76). OGA is required for NONO dissociation from the chromatin and degradation but does not affect other NHEJ factors such as KU70/80. As timely degradation of NONO is necessary to facilitate the subsequent loading of repair factors in the next stage, OGA is important for NHEJ by deglycosylation of NONO. Using the GFP reporter system, we further demonstrated that Thiamet-G (OGA inhibitor) treatment impairs NHEJ, but not HR, further supporting a role of OGA in NHEJ (76).

Poly(ADP-ribose) glycohydrolase (PARG) specifically hydrolyzes the glycosidic bonds between ADP-ribose units in PAR chains and is the primary de-PARylation enzyme responsible for approximately 90% of de-PARylation activity in the cell (94, 95). Previous studies have demonstrated that PARG facilitates both DSB and SSB repair (96, 97). Recently, we reported that PARG undergoes O-GlcNAcylation at Ser-26, which is crucial for maintaining its nuclear localization and chromatin association (98). O-GlcNAcylation is required for recruiting PARG to DNA lesions and its interaction with proliferating cell nuclear antigen. Furthermore, in hepatocellular carcinoma cells, O-GlcNAcylation of PARG enhances the poly(ADP-ribosyl)ation of DNA damage–binding protein 1 (DDB1) and attenuates its autoubiquitination, leading to the stabilization of DDB1. This stabilization enables DDB1 to degrade its downstream targets, such as c-Myc, thereby promoting hepatocellular carcinoma in mouse xenograft models (98). Because of the limitation of this study, the effect on HR or NHEJ of PARG-S26A is not explored. In light of the recent report that PARG regulates DNA replication, especially in Okazaki fragment maturation (99), future studies of PARG O-GlcNAcylation in replication are urgently needed.

### O-GlcNAcylation in BER

BER is a critical DNA repair pathway responsible for addressing DNA lesions, including SSBs and oxidative DNA damage. BER also plays a crucial role in mitochondrial DNA (mtDNA) repair. The initiation of BER occurs when a DNA glycosylase recognizes and removes an improperly modified DNA base. Subsequently, endonucleases and/or phosphodiesterases cleave the resulting abasic site, removing the sugar residue, followed by DNA polymerase and DNA ligase completing the repair process (100).

O-GlcNAcylation has emerged as a regulatory mechanism in BER, influencing the activity of key enzymes involved in this pathway, such as 8-oxoguanine-DNA glycosylase 1 (OGG1). OGG1 is the primary DNA glycosylase responsible for repairing the mutagenic DNA lesions 8-hydroxy-2-deoxyguanosine and ring-opened fapyguanine induced by ROS in humans (101). It is worth noting that mtDNA is more susceptible to the damaging effects of ROS compared with nuclear DNA. This increased susceptibility can be attributed to the lack of a well-organized histone-assisted chromatin structure and the direct proximity of the electron transport chain within the mitochondria (102). A study by Cividini *et al.* (103)demonstrated that OGG1 undergoes significant O-GlcNAcylation in diabetic mice, which inhibits OGG1 activity. Consequently, increased levels of 8-hydroxy-2-deoxyguanosine and an accumulation of mtDNA lesions were observed. These findings provide a plausible biochemical mechanism for diabetic cardiomyopathy. Taken together, O-GlcNAcylation plays a regulatory role in BER, impacting the activity of enzymes like OGG1 involved in repairing oxidative DNA damage. This study also suggests a potential link between O-GlcNAcylation, DNA repair, and the development of diabetic cardiomyopathy. Further research is needed to fully elucidate the underlying mechanism and explore the therapeutic implications of targeting O-GlcNAcylation in BER-related diseases.

### O-GlcNAcylation in translesion DNA synthesis

Compared with HR and NHEJ, relatively less is reported on the role of O-GlcNAcylation in translesion synthesis (TLS). TLS is an error-prone DNA damage tolerance pathway that is employed when cells face replication barriers. Low-fidelity DNA polymerases, such as DNA polymerase η (Polη), utilize the damaged DNA as templates to restart DNA synthesis. Although remarkably efficient to overcome replication barriers, especially compared with the high-fidelity polymerases δ and ε, this process is error prone and therefore must be stringently controlled.

We reported in 2017 that O-GlcNAcylation of Polη facilitates TLS to bypass cisplatin-induced lesions as well as causing increased cellular sensitivity to cisplatin (75). Our study demonstrated that Polη is O-GlcNAcylated at Thr-457, which promotes its polyubiquitination at Lys-462 and subsequent removal of Polη from replication forks. Thus, O-GlcNAcylation plays an important role in Polη-mediated TLS and genome integrity (75).

One of our works in submission involves another important ubiquitin E3 ligase in both TLS and HR: Rad18 (personal communications). We found that Rad18 is O-GlcNAcylated at Ser130/Ser164/Thr468, which are important for its recruitment for DNA damage sites, essential for Polη focus formation and UV sensitivity. In addition, Rad18 O-GlcNAcylation is pivotal for its binding with ubiquitin and Rad51C and HR. As we discovered that UV enhances the chromatin binding of OGT (75), we speculate that more chromatin factors may be coordinated by OGT under UV to participate in TLS.

### O-GlcNAcylation in ICL DNA repair

O-GlcNAcylation also regulates ICL repair. High mobility group box 1 (HMGB1) is a nonhistone chromosomal protein involved in various cellular functions. HMGB1 has been shown to function as a cofactor in nucleotide excision repair, enabling error-free repair of psoralen ICLs and UV-induced intrastrand crosslinks (104). HMGB1 has been identified as a substrate for O-GlcNAcylation, specifically at Ser-100 and 1074. The primary modification site is Ser-100, which has the potential to influence HMGB1–DNA interaction. O-GlcNAcylation reduces the ability of HMGB1 to facilitate DNA repair, leading to error-prone processing of damaged DNA, suggesting a possible connection between elevated O-GlcNAc levels and increased mutation rates in certain cancer conditions (105). This example contradicts most previous studies that have shown O-GlcNAcylation to promote DDR. More in-depth studies are needed. It is possible that O-GlcNAc has distinct roles in different DDR pathways. Alternatively, O-GlcNAcylation of discrete substrates has disparate consequences to coordinate the various steps of DDR.

## Outlooks

In this review, we tried to provide a glimpse of the role of O-GlcNAc in DDR. Although the chances of new DNA repair and damage tolerance mechanisms yet to be discovered are small (106), how OGT–OGA regulate DDR or how OGT–OGA are regulated by DDR remains a wide-open field. Are there other O-GlcNAcylated substrates in HR, NHEJ, BER, or TLS? Are there more substrates in other DDR pathways, such as SSB repair, DNA break-induced repair, or mitotic DNA synthesis? We have no clue. On the other side of the coin, how does OGT or OGA sense the DNA damage signal and be recruited to the damage sites? Are there any PTMs involved? Or is it mediated by protein–protein interactions? Structurally, does recruitment of OGT and OGA to the chromatin entail any conformational alterations? Why does it take longer for OGA to be recruited than OGT? We have no answer either. O-GlcNAc is known as a nutrient sensor, so does DDR respond to distinct nutrient stimuli *via* O-GlcNAc? As O-GlcNAc is most abundant in the brain and pancreas, does it necessitate tissue specificity of O-GlcNAc in DDR? These are all tantalizing questions that await answering. Only by delving deeper into the molecular mechanisms of O-GlcNAc biology, can we utilize the newly developed chemical tools, such as RNA aptamers (107) and nanobody-fused OGT–OGA (108, 109), to their full potential in both research and clinical settings.

## Conflict of interest

The authors declare that they have no conflicts of interest with the contents of this article.
